# The Comparison of Different Types of Heat Accumulators and Benefits of Their Use in Horticulture

**DOI:** 10.3390/s20051417

**Published:** 2020-03-05

**Authors:** Sławomir Kurpaska, Jarosław Knaga, Hubert Latała, Michał Cupiał, Paweł Konopacki, Ryszard Hołownicki

**Affiliations:** 1 Faculty of Production Engineering and Energetics, University of Agriculture in Krakow, ul. Balicka 116B, 30-149 Kraków, Poland; rtkurpas@cyf-kr.edu.pl (S.K.); jaroslaw.knaga@ur.krakow.pl (J.K.); hubert.latala@urk.edu.pl (H.L.); 2 Research Institute of Horticulture, ul. Konstytucji 3 Maja 1/3, 96-100 Skierniewice, Poland; Pawel.Konopacki@inhort.pl (P.K.); ryszard.holownicki@inhort.pl (R.H.)

**Keywords:** accumulators, plastic tunnel, energy, efficiency, sensor

## Abstract

This paper presents the results of the analysis of thermal issues and energy efficiency of three types of accumulators; namely stone-bed; water and phase change. Research experiments were carried out during April–October 2013 in a standard commercial semi-cylindrical high plastic tunnel with tomato cultivation of 150 m^2^. A stone-bed accumulator; with an area of almost 75 m^2^ was installed in the tunnel below ground level; while a water accumulator with a volume of 4 m^3^ was installed outside the tunnel. A phase change material (PCM) accumulator, with a volume of 1 m^3^ containing paraffin, was located inside the tunnel. The heat storage capacity of the tested accumulators and the energy efficiency of the process were determined based on the analyses of the 392 stone-bed charging and discharging cycles, the 62 water accumulator charging cycles and close to 40 PCM accumulator charging and discharging cycles. Dependencies in the form of easily measurable parameters; have been established to determine the amount of stored heat; as well as the conditions for which the effectiveness of these processes reaches the highest value. The presented analysis falls under the pro-ecological scope of replacing fossil fuels with renewable energy. As a result of the analysis; it was found that; in the case of a stone-bed; such an accumulator shows higher efficiency at lower parameters; that is, temperature difference and solar radiation intensity. In turn; a higher temperature difference and a higher value of solar radiation intensity are required for the water accumulator. The energy storage efficiency of the PCM accumulator is emphatically smaller and not comparable with either the stone-bed or the water accumulator.

## 1. Introduction

Currently, the overwhelming majority of the world’s energy comes from non-renewable sources, such as oil, coal, natural gas and uranium, which causes greenhouse gas emissions, global warming and related climate change. Counteracting these adverse phenomena is a huge challenge for modern science and the economy, resulting in the need to conduct research into the possibility of using renewable energy sources as alternatives [1,2,3], with solar energy and methods of its acquisition, processing and storage being the most promising [4]. The high potential for the use of heat obtained from solar energy results from the high efficiency of storage and conversion of this energy [5]. The basic elements used in this field are solar collectors and heat storage systems. Scientific research, conducted for many years, has been aimed at developing appropriate accumulators that will allow energy storage and will be characterized by high efficiency [6,7,8]. Depending on the requirements of the recipient, energy obtained from renewable sources can be converted into various energy types [9]. On the other hand, heat storage is very important in the case of households and farm heating [10], with preparation of domestic hot water for households and farms and food processing being important factors.

### 1.1. Methods of Heat Storage

Among the various methods of storing heat from solar energy, we can distinguish the accumulation of sensible heat [11], latent heat [12] and thermochemical energy [13]. The efficiency of thermal energy storage (TES) systems depends on the properties of selected heat storage materials, the conditions in which these devices are used and the purpose for which they are used [14].

In the case of materials for storing thermal energy in the form of sensible heat, there is no phase change during the process of absorbing thermal energy and the temperature of the medium filling the accumulator increases [15]. The amount of heat stored is proportional to the density, volume, specific heat and temperature changes of the stored material [16]. Heat storage media can be various substances, such as liquids (water, mineral oils, salt solutions, liquid metals and alloys) or solids (stones, concrete, sand, bricks) [17,18,19].

Materials for latent heat storage are phase change materials (PCMs). During the process of absorbing thermal energy, a phase change occurs, and temperature fluctuations are relatively small [20]. These materials use a solid-liquid phase change process. Although the phase transition from liquid to gas is characterized by higher latent heat, large changes in the volume of gases associated with evaporation mean that storage is rarely used in heat accumulators [21]. Materials that use solid-liquid transformation do not have this disadvantage. Such materials should have high latent heat and high thermal conductivity, with a melting point close to the required range of system operating temperatures, should be chemically stable, cheap, non-toxic and non-aggressive [22]. Among the materials using phase change, organic materials (paraffin, fatty acids, esters, alcohols, glycol), inorganic substances (salt solutions, metals and metal alloys, multi-component materials), composite materials and other [14] can be distinguished. PCMs are widely used in heat accumulators [23] and are considered one of the most effective thermal energy storage systems [24,25].

### 1.2. Heat Storage in Protected Crop Cultivation

Protected crop cultivation has been using solar energy for many years. A greenhouse or a plastic tunnel is a closed structure that captures solar radiation and stores heat to create a microclimate that is favorable for plants [26]. The conditions necessary for production of the indoor microclimate depend on the type of crop but as a rule, to maintain the required cultivation temperature, it is necessary to provide additional energy during certain periods [27]. To ensure suitable conditions for growing plants in season as well as out of season, it is very important to store heat in greenhouse production, when the daytime temperature often rises above what is desired and at night may fall below an acceptable temperature [28,29,30]. The systems used in greenhouses should therefore store excess heat accumulated during the day and warm the air inside the facility at night [31]. The strategy for improving the thermal environment in greenhouses must first of all take into account the use of an appropriate energy storage method [32,33].

### 1.3. The Importance of Heat Storage Research 

Appropriate energy management is not limited to effective ways of obtaining it. The occurrence of large fluctuations in energy demand in various branches of the economy makes it very important to develop consistently better energy storage methods [34,35]. This topic is very important for both developed and developing countries. Efficient storage of energy obtained from renewable sources has a positive impact on the natural environment and is an important element of sustainable development. The implementation of appropriate technical and technological solutions in this area should be included in the energy policy of individual countries. Research has shown that governments of countries with a higher share of renewable energy invest more in energy storage technologies [36]. Rational economic energy policy is not only effective processing of renewable energy but also its efficient storage.

The subject of the presented research concerns current issues of energy policy related to food production [37,38,39]. Conventional agricultural production technologies, including protected cultivation, based on non-renewable energy sources, consume around 50 kg of coal per 1 m^2^ of each facility per year in central Europe. As a consequence, the use of outdated, non-ecological technologies can have a very negative impact on energy consumption, as well as on the natural environment [40,41]. The conducted research regarding various methods of heat storage will contribute to the development of optimal solutions dedicated to horticultural production, taking into account the specificity in which heat accumulators are used. The implementation of heat collection and storage technology appropriate in the given conditions is very important both in the context of energy management of individual countries [42], as well as for food production and preservation of natural resources [43]. As thermal issues of heat accumulators are the main problem analyzed in this paper, its main purpose is to review the efficiency of low-temperature heat storage in accumulators that can be used in an engineering practice. As a measure of storage efficiency, the relation between the heat obtained and the electricity used to drive the necessary devices that are an integral part of the analyzed heat storage systems was adopted.

## 2. Materials and Methods

**Figure 1:**
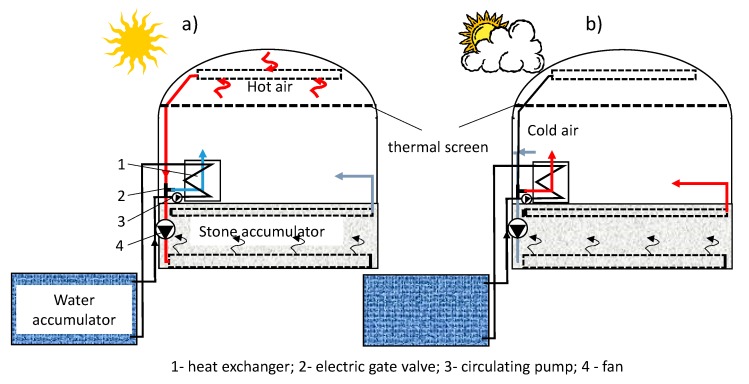
Schematic diagram of the analyzed stone-bed and water accumulator system during the charging (**a**) and discharging cycle (**b**).

The aim of the study was to analyze thermal issues and compare the efficiency of different types of heat accumulators. The research involved three short-term energy storage systems—a stone-bed accumulator, a water accumulator and a PCM (paraffinic) accumulator. The tests were conducted in real conditions at the Faculty of Production Engineering and Energetics in Krakow (19°57’E; 50°03’N) over a 7-month period (April-October). The diagram (Figure 1) presents the idea of a stone-bed and water accumulator during charge and discharge cycles. 

**Figure 2:**
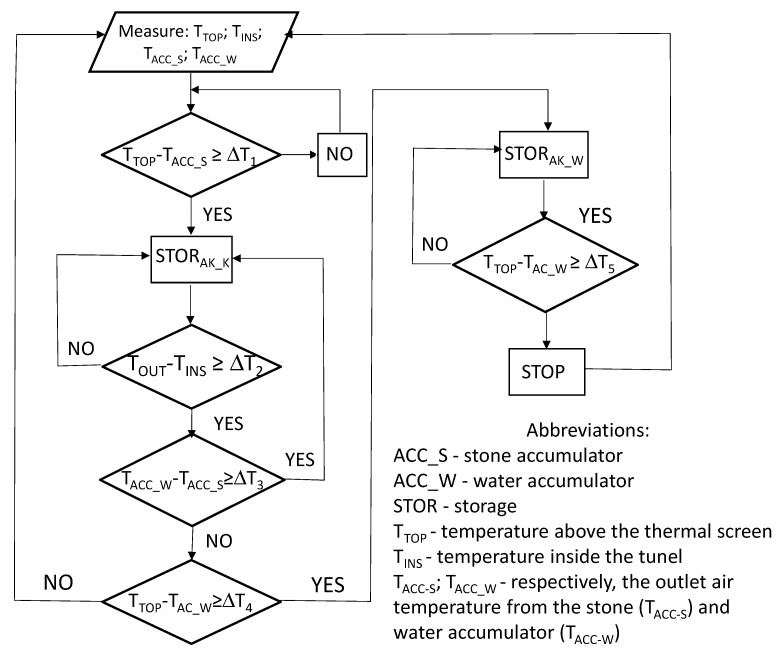
The algorithm diagram for the charging process of the stone-bed and water accumulators.

The charging and discharging processes of both accumulators were carried out in accordance with predefined algorithms. The algorithm diagram for the charging process is shown in Figure 2.

When charging a stone-bed accumulator, it is necessary to provide information about the temperature differences between the temperature above the shading screen and the temperature in the accumulator (ΔT_1_), the temperature at the air outlet of the accumulator and the temperature inside the building (ΔT_2_), as well as the difference between the temperature of the stone-bed accumulator and the temperature of the water accumulator (ΔT_3_). In turn, when charging a water accumulator, it is necessary to know the difference between the air temperature above the shading screen and the initial temperature of the liquid stored in the water accumulator (ΔT_4_) and the water temperature at the end of the charging process (ΔT_5_), therefore tests were carried out with the following predefined threshold values of the differences of the following parameters—ΔT_1_ = ΔT_4_ = ΔT_5_ = 4 K; ΔT_2_ = ΔT_3_ = 2 K. The discharge process of the stone-bed accumulator was carried out using a similar algorithm in which it was assumed that the air flow was forced through the stone-bed when the temperature difference between the temperature of the air flowing out of the accumulator and the temperature inside the building was at least 2 K. 

### 2.1. The Stone-Bed Accumulator

**Figure 3:**
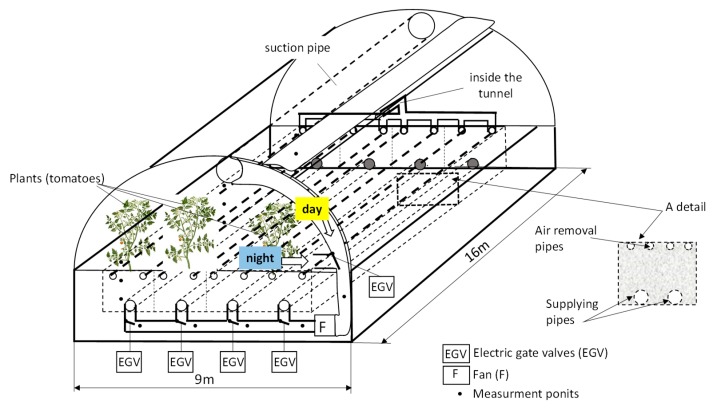
A diagram of an experimental direct contact ground-air heat exchanger located below a semi-cylindrical high plastic tunnel.

**Figure 4:**
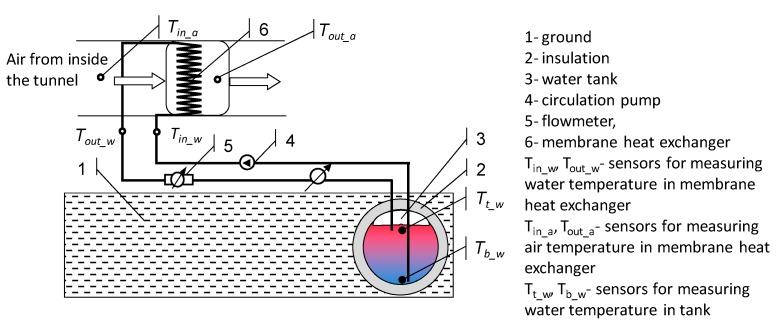
Diagram of the stand for measuring the efficiency of the water accumulator.

**Figure 5:**
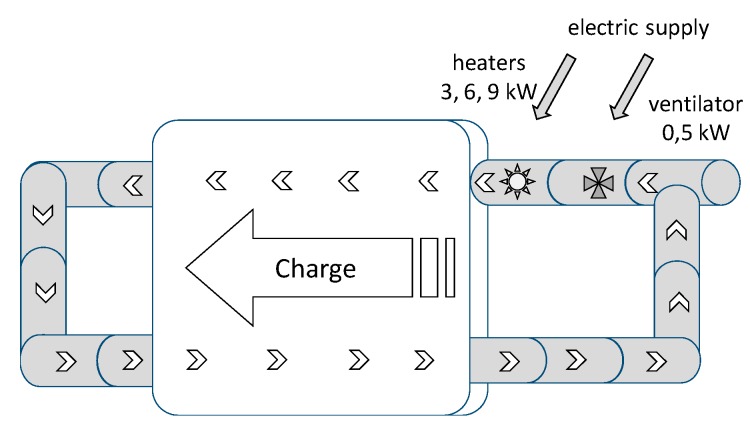
Charging the phase change material (PCM) accumulator in a closed distribution of a hot air.

The diagram (Figure 3) presents the heat storage system in the stone-bed accumulator, while the diagrams (Figure 4 and Figure 5) present the heat storage system in the other analyzed accumulators. Tomatoes (with a density of 2.1 plants/m^2^) were grown during the experiment. 

The stone-bed accumulator comprised two independent sections with dimensions (length × width)—11 × 1.7 m and 11 × 3.4 m, respectively. Each section has a deposit height of 0.7 m and was separated from the other by means of an insulation layer. The stone-bed accumulator deposit was porphyry particles (fraction 30–60 mm). The porphyry used was characterized by the following parameters—thermal conductivity coefficient 1.67 W/(m∙K); bulk density 2550 kg/m^3^ and in the further analysis the equivalent particle diameter was assumed to be 0.045 m. Perforated pipes supplying air were located in the lower part of each section, while the perforated pipes removing the air were located in the upper part of each section. The air was forced from the ducts into the tunnel. The system employed a fan powered by an electric engine with a power rating of 1.8 kW. The motor was connected directly to a variable frequency controller, matched to the engine power, which allowed modulation of the speed of air flowing through the accumulator. The experiments were carried out for various accumulator surfaces ranging from 18.7 m^2^ to 74.8 m^2^, while in order to unify the obtained results, all analyses were carried out for the unit area and the unit time of the experiment. A total of 392 cycles were analyzed, split equally between charging and discharging. During the experiments, the following parameters were measured—air flow velocity (V_a_) measured in a measuring section with a diameter of 300 mm—using an air flow meter MiniAir64, air temperature—using a PT 1000 resistance sensor and relative humidity—using a HD4917T meter. The measurement accuracies were for temperatures from ±0.35 to ±0.55 K and for humidity and air flow ±1.5%. Whereas the total solar radiation intensity R_out_ to the horizontal surface and the matching one from the tunnel was measured with a CM3 pyranometer. All measurements were monitored and archived using the author’s own measuring system with a sampling time of 120 s.

### 2.2. The Water Accumulator

The system built for testing was equipped with an air-water heat exchanger with a power rating of 12 kW. The heat exchanger system used a fan powered by an electric engine with a power rating of 3 kW. The engine was connected directly to a variable frequency controller, matched to the engine power, which allowed modulation of the speed of air flowing through the exchanger. The entire air-to-water heat exchanger system was connected by a hydraulic system with a water heat accumulator of 4 m^3^ volume. The experimental water heat storage tank was made on the basis of a tank with a total capacity of 5 m^3^ in the shape of a cylinder with a diameter of 1.6 m and a length of 2.5 m. The exterior layer of the tank consisted of thermal insulation, in the form of 10 cm thick extruded polystyrene foam, with any gaps between the polystyrene foam plates filled with polyurethane foam. As a circulating pump, a submersible pump was used, in order to facilitate venting the system without any problems, the construction of which is based on the principle of a hydraulic jack. The circulation force of the pump was 300 W. The design of the heat storage system is shown in the diagram (Figure 4).

In order to measure the energy and power of the heat source, resistive Pt 100 sensors were used and for measuring temperature at other points Pt 100 and Pt 1000 sensors were employed. The flow rate of the fluid (water) was measured using a water meter equipped with a pulse transmitter GMDX-R. The volume per each pulse in said flow meter was 10 dm^3^. Electricity consumption was measured with an LE-03 active power meter in the first accuracy class. The measuring instruments used met the quality requirements of laboratory tests. The operating parameters of the system were recorded at a predefined time interval of 120 s and at each additional event. 

### 2.3. The Phase Change (PCM) Accumulator

The phase change (PCM) accumulator was filled with 1 m^3^ of R58 paraffin. The accumulator’s construction was a rectangular heat exchanger, which contained a pipe with a diameter of 100 mm, through which warm or cool air flowed. Heat transfer or collection from air took place through the walls of the exchanger pipes to the paraffin. Accumulation of solar radiation energy in this accumulator was analyzed in cycles. They included variable time of energy supply to the accumulator, stagnation period and variable discharge time. The supply of energy for accumulation took place in the process of solar radiation conversion in a photovoltaic power plant. This energy was then converted into heat employing a set of heaters with 3, 6 and 9 kW, respectively and was supplied as a stream of warm air to the accumulator. The work of individual heaters depended on the available instantaneous power of the solar power plant. The assumption of the experiment was to use the potential of available solar energy and its utilization through the charging process. That is, on the one hand, how much heat can be supplied to the accumulator and on the other hand, how much heat can be absorbed by the material filling the accumulator. After the charging period, the accumulator stagnated until the discharge cycle started. In the discharge process, useful heat was transferred directly with a stream of air returned to the tunnel. During the experiment, the following parameters were measured:

(1) at the input—working time, heating energy for the heaters and the fan, paraffin temperature, temperature and relative humidity of the air entering the battery.
(2) at the output—working time, fan power energy, paraffin temperature, temperature and relative humidity of the air inlet and outlet from the battery.

The diagram (Figure 5) shows the process of charging the PCM accumulator. Heaters supplying heat to the accumulator were switched on sequentially depending on the available power of a photovoltaic power plant, while a 0.5 kW axial fan was responsible for the distribution of the heat flux in the air.

**Figure 6:**
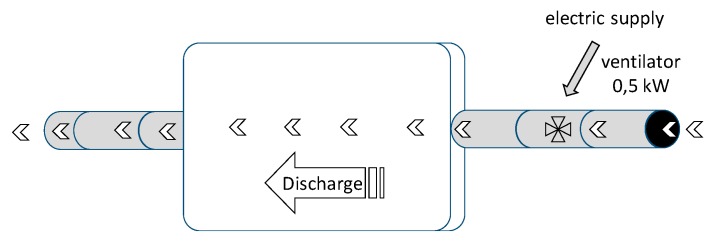
Discharging the PCM accumulator using a stream of air.

The process of discharging the PCM accumulator (Figure 6) consisted of receiving heat accumulated in the paraffin by means of an air stream flowing through the bed. For this purpose, the fan used earlier in the accumulator charging process was employed. The useful heat in this process was the difference between the air enthalpy at the output and the input of the PCM accumulator. 

### 2.4. Methods

Comparison of individual systems for heat collection and short-term storage allows the determination of preferences in the application of the selected solution for individual conditions in which agricultural production is carried out. This choice has a significant impact on energy policy with regard to the replacement of conventional energy sources in horticultural production with renewable energy. Storage efficiency, calculated as the energy effect obtained in relation to the expenditure incurred, was adopted as an indicator of the usefulness of the analyzed accumulators. 

#### 2.4.1. Stone-Bed Accumulator

**Figure 7:**
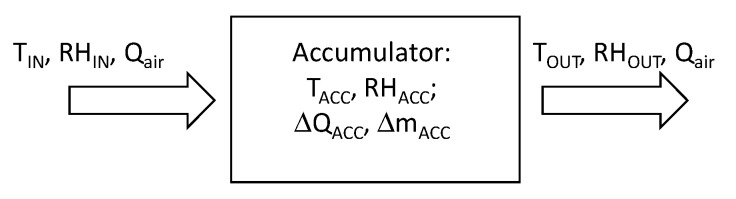
Input and output fluxes with processes occurring in the accumulator bed.

The intensity of heat and mass exchange processes in the accumulator depends on variations in temperature (T_IN_) and humidity (RH_IN_) and the flux of injected air (Q_air_), as well as temperature (T_ACC_) and humidity inside the accumulator (RH_ACC_). The diagram (Figure 7) presents input and output parameters that allow full analysis of the processes taking place in the accumulator bed.

The process presented in Figure 7 covers two issues (interconnected), namely heat exchange (between flowing air and bed particles) and mass exchange (as a result of evaporation/condensation processes). The general equation of mass energy balance for air contained in the accumulator bed was formulated as an equation with concentrated parameters, taking into account streams inflowing or out-flowing from the analyzed space. Hence, taking into account the heat and mass balance equations, in differential time dτ, one can arrive at the following conclusions:

for heat:

(1) $${\mathrm{dQ}}_{ACC}=V_{a}\cdot F\cdot \rho_{a}\int_{\tau_{1}}^{\tau_{1}+\tau }\left(H_{IN}-H_{OUT}\right)d\tau ,$$

where *Q_ACC_*—heat in the accumulator (kWh); *V_a_*—air velocity in the measuring section (m/s); *F*—cross-sectional area of the section in which the velocity was measured (m^2^); *ρ_a_*—air density (kg/m^3^); *m_ACC_*—change in the water mass in the accumulator (kg); *H_IN_*, *H_OUT_*—enthalpy of air forced in (*H_IN_*) and flowing out of the accumulator (*H_OUT_*), kWh/kg.

and for mass:

(2) $${\mathrm{dm}}_{ACC}=V_{a}\int_{\tau_{1}}^{\tau_{1}+\tau }\left(C_{IN}-C_{OUT}\right)d\tau ,$$

where *m_ACC_*—change in the water mass in the accumulator (kg); *C_IN_*, *C_OUT_*—absolute humidity in the air forced in (*C_IN_*) and flowing out of the accumulator (*C_OUT_*) (kg/m^3^).

When considering heat and mass issues, all parameters were monitored and archived by a computer measuring system with a recording frequency every 120 s. To generalize the results obtained, the average values of the measured air parameters (*W_avg_*) determining the changes in heat and mass of water in the accumulator bed were calculated during subsequent work cycles (charging and discharging). These quantities were calculated on the basis of their instantaneous values according to the relationship:

(3) $$W_{avg}=\frac{1}{\tau }\int_{\tau_{1}}^{\tau_{1}+\tau }w\left(\tau \right)d\tau .$$

Storage efficiency (Coefficient of Performance) was determined for three cases, namely the charging process, the discharging process and as a summary effect of the charging and discharging processes together. It can therefore be formally recorded that the efficiency for the loading process (*COP_acc_ch_*) was:

(4) $$COP_{acc\_ch}=\;\frac{\sum_{\tau =0}^{\tau =\tau_{1}}\left(Q_{OUT}-Q_{IN}\right)}{P_{e\_ch}\cdot \tau_{1\_ch}},$$

where *Q_OUT_*, *Q_IN_*—heat inflow (*Q_IN_*) and outflow (*Q_OUT_*) from the accumulator (kWh); *P_e_ch_*,—power of the fan engine in the process (kW), *τ_1_ch_*_—_accumulator charging time (hr).

In turn, for the discharge process, process efficiency (*COP_acc_disch_*) was calculated as:

(5) $$COP_{acc\_disch}=\;\frac{\sum_{\tau =0}^{\tau =\tau_{1}}\left(Q_{IN}-Q_{OUT}\right)}{P_{e\_disch}\cdot \tau_{1\_disch}},$$

where *P_e_disch_*,—power of the fan engine in the process (kW), *τ_1_disch_*—accumulator discharging time (hr).

Finally, the efficiency of the entire process (*COP_tot_*) was described using the formula:

(6) $$COP_{acc\_tot}=\;\frac{\sum_{\tau =0}^{\tau =\tau_{1}}\left(Q_{IN}-Q_{OUT}\right)}{P_{e\_ch}\cdot \tau_{1\_ch}+P_{e\_disch}\cdot \tau_{1\_disch}}.$$

Other parameters necessary to determine these relationships for each cycle (enthalpy—*H*; absolute air humidity—*C*) were calculated using psychrometric relationships. In order to compare the observed changes in environmental conditions inside the tunnels studied, the water vapor pressure deficit (vpd) was also calculated for the air in tunnels with and without a accumulator (control tunnel). In both tunnels, the same density of plants was used during the experiments and the construction of tunnels and control of the position of roof vents were identical.

#### 2.4.2. Water Accumulator

For a 2-min interval, the amount of heat stored in the water accumulator was determined in accordance with the relationship (7):

(7) $$Q=\frac{1}{3.6}\int_{V_{i}}^{V_{i+1}}\rho \cdot c_{w}\cdot \left(T_{out\_w}-T_{in\_w}\right)dV,$$

where *Q*—heat (kWh); *dV*—change in the volume of flowing working medium (dm^3^); *ρ*—working medium (water) density (kg/dm^3^); *c_w_*—specific heat of the working medium (kWh/(kg⋅K)); *T_in_w_*—system inlet temperature (°C); *T_out_w_*—system outlet temperature (°C).

However, when operating in a steady-state, the inlet temperature from the exchanger changes towards the temperature at the bottom of the accumulator *T_in_w_*→*T_b_w_* and the temperature of the upper zone of the tank changes towards the temperature at the outlet of the exchanger *T_t_w_*→*T_out_w_*. The average temperature in the water heat accumulator *T_B_* will be calculated as the average value from the temperatures of the upper *T_t_w_* zone and the lower *T_b_w_* zone of the battery. 

Based on the relationship (7), the instantaneous power of the heat stream forced into the accumulator was determined in accordance with the relationship (8):

(8) $$\dot{Q}=\frac{dQ}{d\tau },$$

where *dQ*—heat (kWh); *dτ*—time when the heat was accumulated *dQ* (hrs).

For the needs of system dynamics analysis, the instantaneous heat storage efficiency was determined in accordance with the relationship (9):

(9) $$COP_{w}=\;\frac{\dot{Q}}{P_{e}}\;,$$

where $\dot{Q}$—heat stream (kW); *P_e_*—electric power recorded by the analyzer (kW).

The last element of the energy efficiency analysis of the heat storage system is the efficiency related to the system’s work cycle. Periods of uninterrupted operation with a positive heat balance of a duration not shorter than 2 h and not longer than the time of the daily interval were assumed as the cycle of operation of the heat storage system in a water accumulator. The energy efficiency defined in this way was determined according to the relationship (10):

(10) $$COP=\frac{\sum_{\tau =0}^{\tau =\tau_{k}}Q_{i}}{\int_{\tau_{0}}^{\tau_{k}}P_{e}d\tau }$$

and after reducing to a common base of time the relationship (11) was obtained:

(11) $$COP=\frac{\int_{\tau_{0}}^{\tau_{1}}\dot{Q}d\tau }{\int_{\tau_{0}}^{\tau_{1}}P_{e}d\tau },$$

where $\dot{Q}$—heat stream (kW); *τ_0_*—heat storage system activation time (s), *τ_k_*—heat storage system shutdown time (s), *P_e_*—electric power recorded by the analyzer (kW).

#### 2.4.3. PCM Accumulator

**Figure 8:**
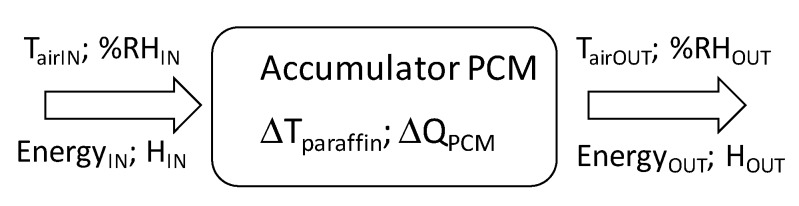
Parameters at the input and output of the PCM accumulator.

During the charging process, heat to the PCM accumulator (Figure 8) was supplied by means of an air stream heated by resistance heaters (Energy _IN_). As a result, the paraffin temperature (ΔT_paraffin_) and internal energy of the bed (ΔQ_ACC_) increased. Reception of heat from the accumulator (discharge cycle) was carried out using cool air stream (T_airIN_) heating (T_airOUT_) in the accumulator bed. The difference in paraffin temperature was calculated for each cycle of accumulator charging and discharging, thus determining how the temperature increased or decreased in a given cycle.

The general energy balance equation in the accumulator bed was formulated in the form of Equations (12)–(14) taking into account the inflows or outflows from the analyzed bed. Hence, taking into account the heat balance equations in differential time dτ, one can write:

(12) $${\mathrm{dQ}}_{PCM}=\left({d\dot{Q}}_{HEAT}\;-{d\dot{Q}}_{E}\right)d\tau$$

(13) $${\mathrm{dQ}}_{HEAT}=\;\int_{\tau_{1}}^{\tau_{1}+\tau }P_{HEAT}d\tau$$

(14) $${\mathrm{dQ}}_{E}=\int_{\tau_{1}}^{\tau_{1}+\tau }\left({d\dot{Q}}_{OUT}\;-{d\dot{Q}}_{IN}\right)d\tau ,$$

where *Q_PCM_*—heat in PCM accumulator (kWh); *P_HEAT_*—heater power (kW); ${\dot{Q}}_{IN},\;{\dot{Q}}_{OUT}$—energy of stream of air forced in and flowing out of the accumulator (kW); $\dot{Q_{e}}$—useful energy stream (kW); ${\;\dot{Q}}_{HEAT}$—supplied energy stream (kW).

The energy efficiency of the heat storage system in the phase change material was referenced to the system’s work cycle. The energy efficiency of the PCM accumulator was determined according to the relationship (15):

(15) $$COP=\frac{\int_{\tau_{0}}^{\tau_{1}}\dot{Q_{e}}\;d\tau }{\int_{\tau_{0}}^{\tau_{1}}P_{e}\;d\tau +\int_{\tau_{0}}^{\tau_{1}}{\dot{Q}}_{IN}\;d\tau }\;,$$

where $\dot{Q_{e}}$—useful heat stream (kW); *t_0_*—heat storage system activation time (s); *t_k_*—heat storage system shutdown time (s); *P_e_*—electric power recorded by the analyzer (kW); $\dot{Q_{IN}}$—input air heat flow (kW).

## 3. Results

### 3.1. Stone-Bed Accumulator

Heat storage efficiency tests were carried out at the following input parameter ranges. For accumulator charging, 0.1 < *τ* < 23.6 hrs; 11.5 < *T_INS_* < 29.3 °C; 23.1 < RH_INS_ < 90.9%; 3.7 < *V_a_* < 6.1 m/s, 470 < *P_e_ch_* < 1180 W. For accumulator discharging, 0.2 < *τ* < 20.1 hrs; 6.6 < *T_INS_* < 22.2 °C; 73.1 < RH_INS_ < 95.2 %; 4.6 < V_a_ < 6.4 m/s, 830 < *P_e_disch_* < 1250 W. 

The PCM accumulator system operation included charging and discharging periods. Charging times ranged from 1.9 to 10.3 h and discharging times ranged from 2.5 to 11 h.

**Figure 9:**
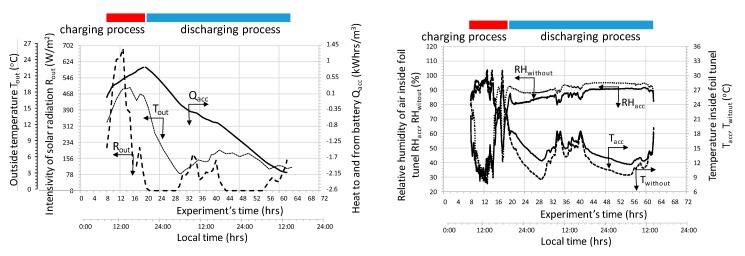
The course of measured and calculated parameters during a full cycle of charging and discharging the stone-bed accumulator.

Figure 9 shows an example of the changes of measured and calculated parameters during one full cycle of accumulator charging and discharging. 

During the charging cycle, the total amount of solar radiation energy was 4.17 kWh. Whilst charging (the cycle time was nearly 11 hours), the total amount of heat stored in the accumulator (calculated per 1m^3^ of the bed) was 0.84 kWh/m^3^. In the discharge cycle (cycle duration was approximately 43 h), about 2.9 kWh / m^3^ was fed into the tunnel with plants. The greater amount of heat delivered to the tunnel with plants, compared to the amount of heat stored during accumulator charging, was at the expense of lowering the final bed temperature. 

**Figure 10:**
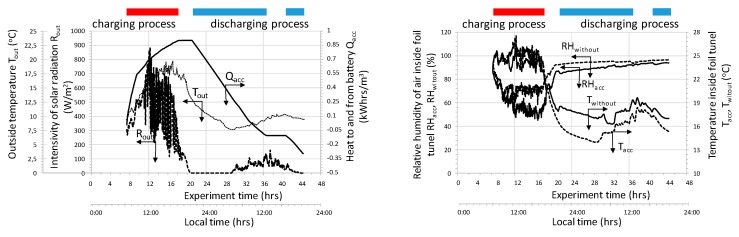
The course of measured and calculated parameters during the full cycle of charging and discharging a stone-bed accumulator.

Figure 10 illustrates the charge and discharge cycles in which the total amount of solar radiation in the cycle was 5.5 kWh.

The charging cycle was approximately 11.5 h. During charging, nearly 0.91 kWh/m^3^ of heat was stored in the accumulator, while during discharge 1.2 kWh/m^3^ of heat was supplied to the tunnel. As before, the increased amount of heat results from a lower bed temperature at the end of accumulator discharge when compared to the onset of charging. 

**Figure 11:**
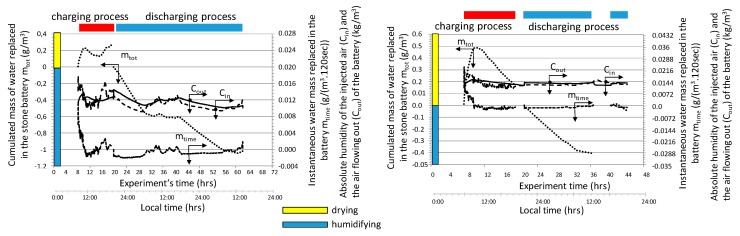
Change in the concentration of water vapor in the air forced in and flowing out of the bed, as well as the instantaneous and total water flow exchanged between the accumulator and the tunnel interior.

As shown in the graph (Figure 11), the air passing through the bed of the accumulator’s bed is dehumidified while the accumulator is being charged and humidified when it is discharged. The total amount of water removed from the interior of the tunnel (*m_tot_*) when charging the accumulator depends on the temperature of the injected air. As it results from the course of the presented relationships at the beginning of the charging process, the amount of water mass stored in the accumulator bed increased intensively, while with the heating of the bed the air drying rate decreases and even to the extent that humidification of exhaust air occurs. This trend was observed for both analyzed cases.

**Figure 12:**
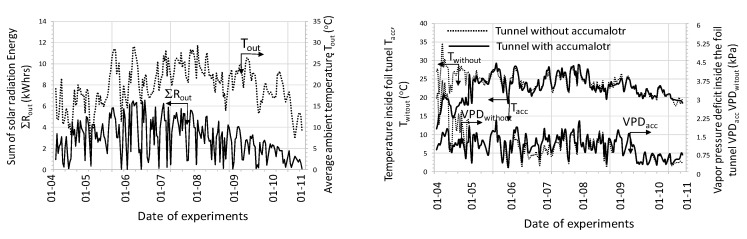
Averaged course of measured parameters during accumulator charging cycles.

**Figure 13:**
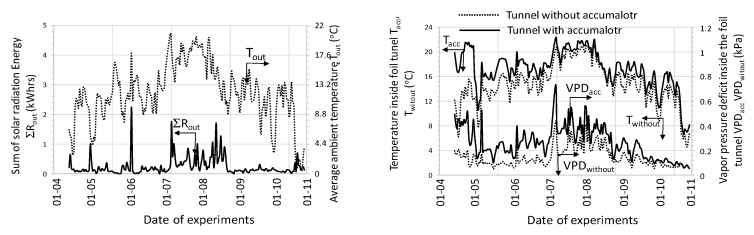
Averaged course of measured parameters during accumulator discharging cycles.

**Figure 14:**
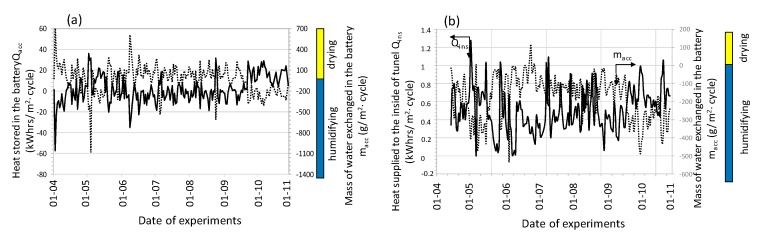
Changes in heat and mass during charge (**a**) and discharge (**b**) cycles.

In order to generalize the relationships obtained, the analyses were carried out for all 392 cycles and the obtained graphical relationships of the measured and calculated quantities were depicted in Figure 12, Figure 13 and Figure 14. 

The analysis showed that throughout the entire season from 0.003 to 1.27 kWh of heat was supplied to the inside of the plastic tunnel (area 150 m^2^) from 1 square meter of accumulator surface during one cycle. Taking into account the accumulator area (the active area in both processes was about 70 m^2^) and the number of cycles, 7.09 MWh of heat was supplied to the interior of the tunnel. This is equivalent to combustion of almost 1.74 tons of coal, assuming a boiler efficiency of 0.7 and a calorific value of 5.83 kWh / kg. During both operations, the process of humidifying and drying the air stream occurred. The average amount of water vapor removed from the flowing air as a result of its condensation at the flowing air-bed interface (in the charging process) was 0.03 kg_H20_/cycle per 1 m^2^ of accumulator surface. When discharged, exhaust air was humidified, and its average value was 0.17 kg_H20_/cycle/m^2^. The mechanism of increasing humidity in the exhaust air results directly from the evaporation of water vapor in contact with air at a lower temperature than the temperature of the stone bed. To sum up, it can be stated that the dominating process during the accumulator charging was dehumidification, while during the discharge process it was humidified. 

**Figure 15:**
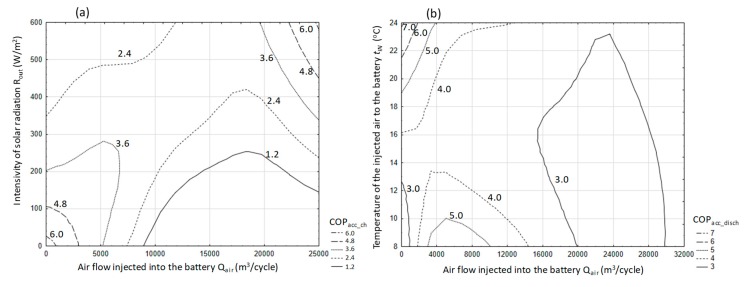
The course of the energy efficiency coefficient of performance (COP) for the process of charging (**a**) and discharging (**b**) a stone-bed accumulator.

Figure 15 presents selected changes in the COP coefficient as a function of solar radiation intensity and the amount of air forced in for the accumulator charging process (a) and as a function of the temperature and amount of air forced in during its discharge (b). 

As can be seen in both cases, when the air stream increases, the efficiency of the processes analyzed decreases. In the examined conditions, the range of changes in the COP coefficient was, for the charging process, from 0.1 to 9.0 and for the discharge process within the range of 0.2 to 9.8.

**Figure 16:**
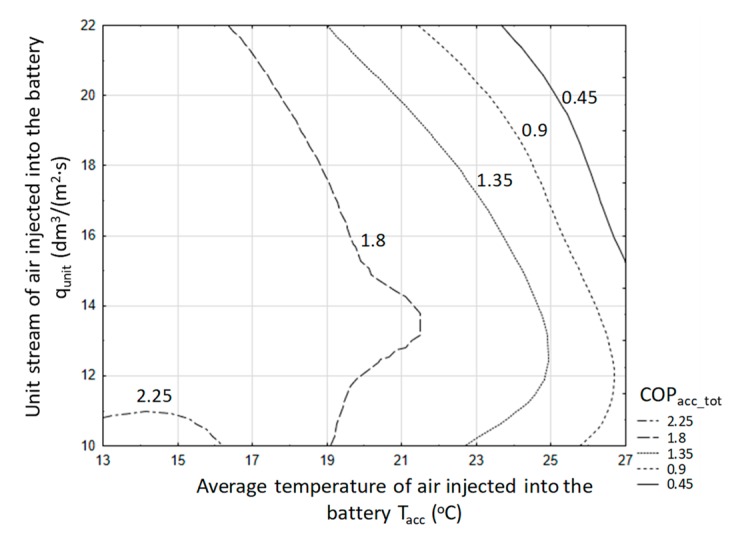
The course of the COP coefficient taking into account the charging and direct discharging of a stone-bed accumulator.

Figure 16 presents the course of the COP coefficient for successive charging and direct discharging cycles (the coefficient was calculated from Equation (6)). 

Research shows that the COP coefficient decreases as the temperature and stream of injected air increase. In the experimental conditions, the average value of this coefficient was COP = 1.71. Based on the results obtained by the non-linear estimation method using the quasi-Newton method, with a significance factor of α = 0.05, an equation was found for the COP coefficient as a function of mean temperature of the injected air and air stream. This equation takes the form:

(16) $$COP=-3.52E^{-11}\cdot T_{in\_avg}^{7.47}+7.6\cdot q_{unit}^{-0.009}-5.31;\;R^{2}=0.88$$

for the ranges of input variables, 13.3 < T_in_avg_ < 25.1 °C; 9.41 < q_unit_ < 21.84 dm^3^/(m^2^∙s), where T_in_avg_—mean temperature of the air forced into the accumulator in the processes of its charging and discharging (°C); q_unit_—stream of injected air per the accumulator surface unit (dm^3^/(m^2^∙s)). 

### 3.2. Water Accumulator

**Figure 17:**
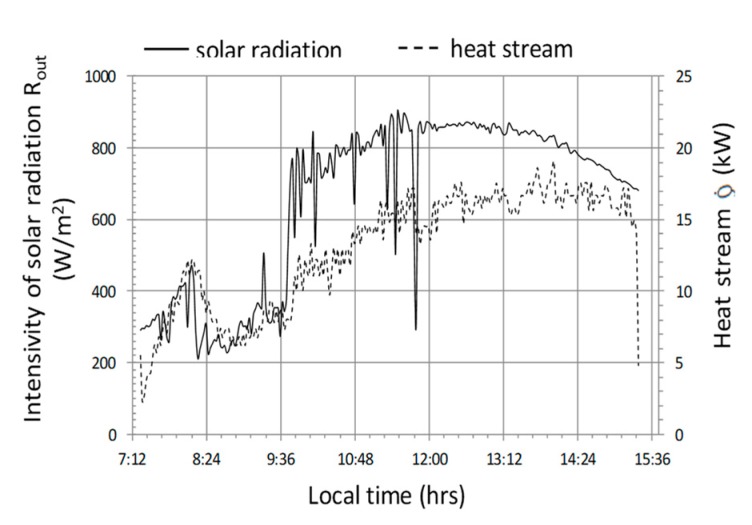
Timescale of solar radiation intensity and heat stream transferred to the accumulator for cycle No. 1.

**Figure 18:**
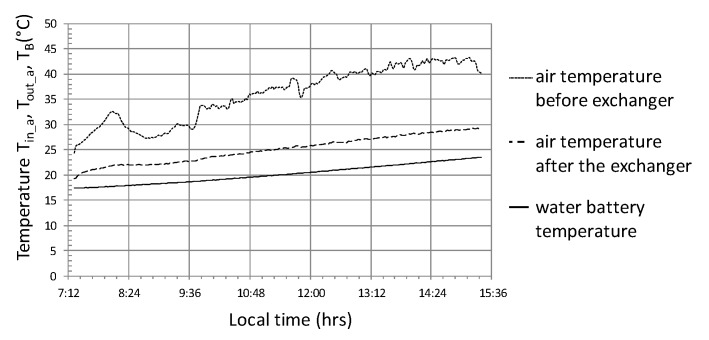
Timescale of temperature distribution in the heat storage system in the water accumulator for cycle No. 1.

The analysis of the test results is presented in graphs for three independent cycles. Figure 17 presents the operating parameters of the analyzed heat storage system for the cycle with the following average values—R_out_a_ = 655 W/m^2^, T_in_aa_ = 36 °C, T_out_aa_ = 26 °C, T_B_a_ = 20.2 °C, ${\dot{Q}}_{a}$ = 13 kW (where, index a stands for the average value of the parameter which was calculated in accordance with equation 3), COP = 4.6. This cycle was chosen because it lasted 8 h and was the longest in the analyzed data set from the period of 7 months. In this cycle, solar radiation reaching the object was stable, without major interference, on a typical cloudless day with a maximum at 12:30 of 860 W/m^2^. The course of the instantaneous heat flow $\dot{Q}$ is correlated with the intensity of solar radiation, which can be clearly seen in the period of operation from 10:00 to 13:00 (Figure 17). The maximum value of this stream is at 14:00 and is 17.5 kW. The end of the system operation in this cycle is characterized by a sharp drop in power, which, in conjunction with the temperature course (Figure 18) may indicate a forced shutdown of the heat storage system in the water accumulator. An important phenomenon is smoothing the temperature of the air leaving the heat exchanger (Figure 18) in relation to the temperature course of the air entering this heat exchanger. The water temperature T_B_ in the accumulator increases from 17.5 to 23 °C, which equates to a storage of 105 kWh of heat (Figure 18).

**Figure 19:**
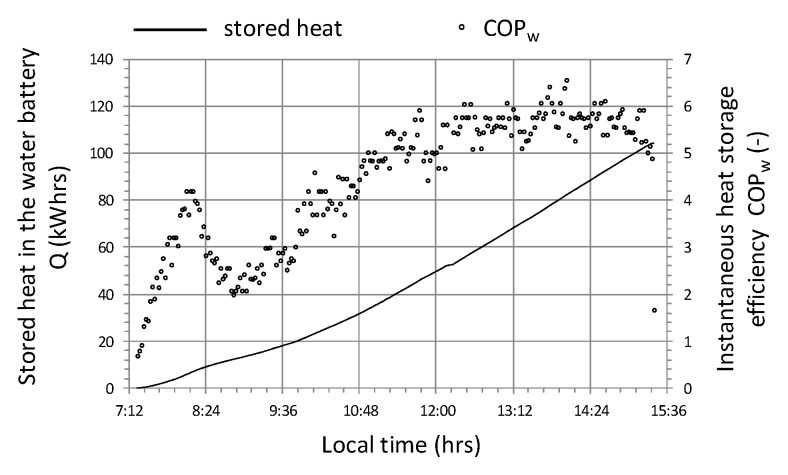
The course of changes in the efficiency of heat storage and the amount of heat stored in cycle No. 1.

Attention should be paid to the course of the instantaneous energy efficiency coefficient (COP_w_) shown in the graph (Figure 19), which in this case is clearly correlated with the intensity of solar radiation R_out_ (Figure 17). Based on the analysis of the presented cycle, it can be concluded that the system should not be turned on if the difference in temperature of air T_in_a_ inside the tunnel and water in the accumulator T_B_ is lower than 10 °C (Figure 17 and Figure 19), because energy efficiency COP_w_ in this range is less than 2. In addition, in this cycle, the maximum energy efficiency of the heat storage system was observed at 6 with a temperature difference (T_in_a_ − T_B_) = 20 and solar radiation intensity R_out_ = 800 W/m^2^.

**Figure 20:**
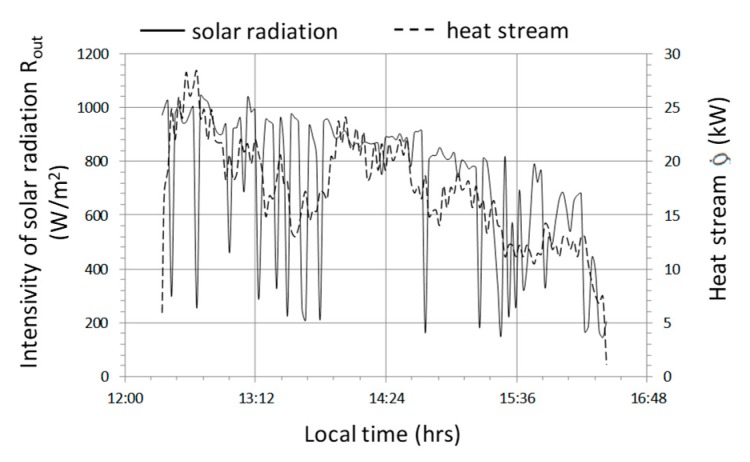
Timescale of solar radiation intensity and heat stream transferred to the accumulator for cycle No. 2.

**Figure 21:**
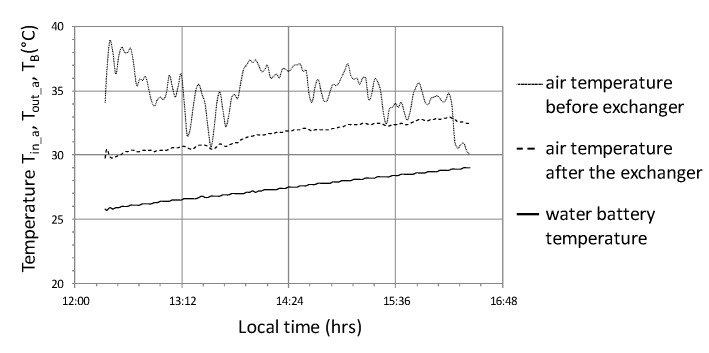
Timescale of temperature distribution in the heat storage system in the water accumulator for cycle No. 2.

**Figure 22:**
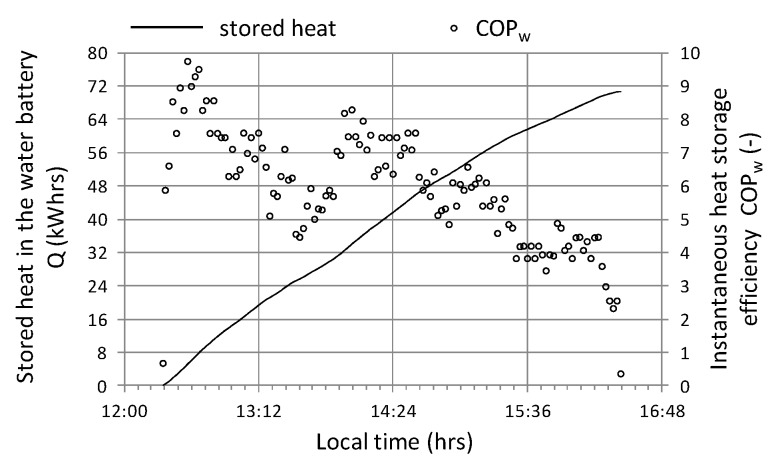
The course of changes in the efficiency of heat storage and the amount of heat stored in cycle No. 2.

The next cycle selected for analysis (No. 2) was characterized by the following average values—R_out_a_ = 730 W/m^2^, T_-in_aa_ = 35 °C, T_out_aa_ = 31.0 °C, T_B_a_ = 27.4 °C, $\dot{Q_{a}\;}$ = 17 kW, COP = 5.96. The charts (Figure 20, Figure 21 and Figure 22) present selected operating parameters of the examined system for a cycle that lasted over 4 hours and was two times shorter than cycle No. 1. In cycle No. 2, solar radiation reaching the tunnel was subject to quite rapid changes at relatively high maximum instantaneous values exceeding 1000 W/m^2^. Temporary short-term cloud cover was present for no longer than a few minutes; however, it was characterized by a significant decrease in solar radiation to the value of 200 W/m^2^ (Figure 20). On the other hand, the course of the instantaneous heat flow $\dot{Q}$ was subject to greater changes in the range of 27 to 10 kW, the effect of which can be noticed in the form of heat impact after switching on the system, which lasted about 15 min (Figure 21) and resulted from the fact that the system was turned on after heating the tunnel additionally during the period of greatest radiation. The time course of the temperature in the water accumulator T_B_ and the temperature of the air leaving the exchanger T_out_a_ steadily increased with rapid changes in the inlet air temperature T_in_a_ (Figure 21).

In the example of temperature courses, one can notice the positive effect of a significant heat capacity of the heat exchanger, which, among others permits operating continuity with rapid changes in solar radiation. The effects of heat shock as well as the high heat capacity of the air-water heat exchanger system are clearly marked on the course of the instantaneous energy efficiency (Figure 22), where values reach the dynamic maximum at level 9 and decrease quite quickly with quite considerable scatter. The graph (Figure 21) shows premature shutdown due to the fact that the inlet temperature T_in_a_ is lower than the temperature of the air outlet T_out_a_ from the heat exchanger, despite the fact that the system works with an efficiency of over 2. 

**Figure 23:**
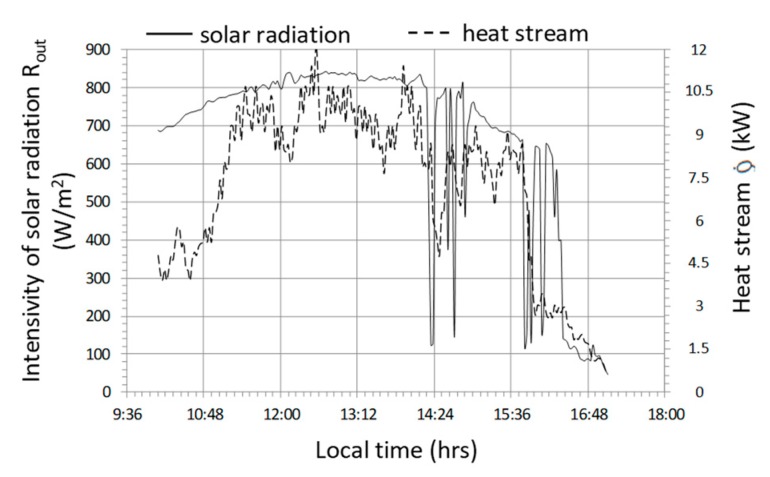
Timescale of solar radiation intensity and heat stream transferred to the accumulator for cycle No. 3.

**Figure 24:**
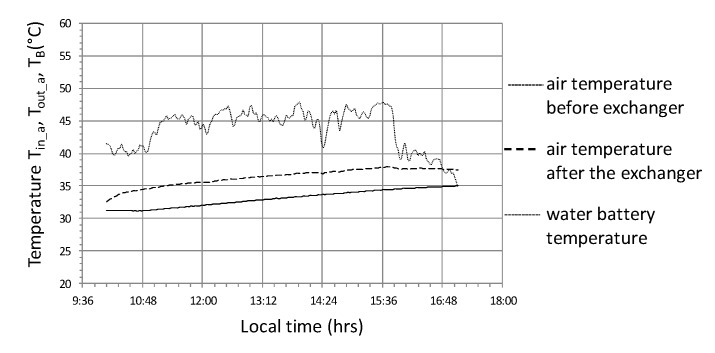
Timescale of temperature distribution in the heat storage system in the water accumulator for cycle No. 3.

**Figure 25:**
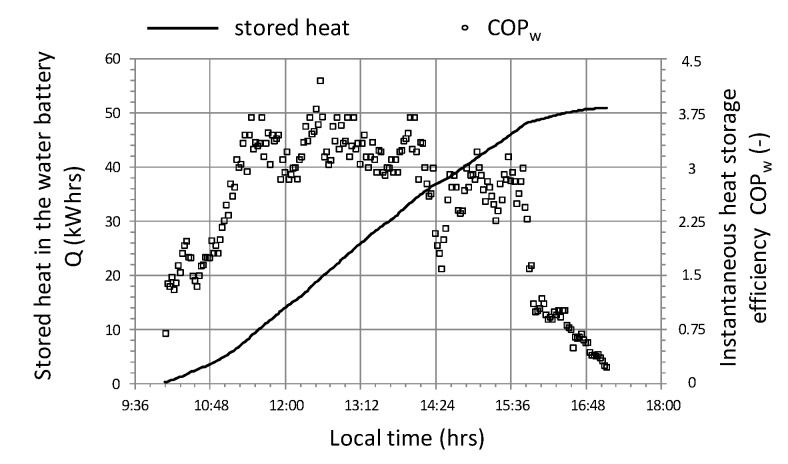
Changes in the efficiency of heat storage and the amount of heat stored in cycle No. 3.

The last cycle selected for detailed analysis as part of the case study is the cycle marked No. 3 (Figure 23, Figure 24 and Figure 25) which is characterized by the following average values of selected parameters—R_out_a_ = 660 W/m^2^, T_in_aa_ = 43.7 °C, T_out_aa_ = 36.4 °C, T_B_a_ = 27.4 °C, $\dot{Q_{a}}$ = 7.2 kW, COP = 2.74. The analyzed cycle No. 3 was selected because of the low energy efficiency with relatively high operating parameters and duration comparable to cycle No. 1 but with a shift of three hours towards the later daytime interval. 

During changes in solar radiation (Figure 23) quite rapid changes of heat flux occur, with a significant reduction in value from 10.5 to 1.5 kW. The system became more sensitive, which was affected by the limited effects of heat capacity. The result of a significant reduction in the thermal efficiency of the system is the relatively high temperature of the water accumulator T_B_, which in this cycle varies between 31.5 ÷ 35 °C (Figure 24). High accumulator temperature also induces high temperatures of other parameters T_in_a_ i T_out_a_ with low differences in these temperatures, for example, T_out_a_—T_B_ is the lowest of the analyzed cycles and does not exceed 3.5 °C. Although the temperature 45 °C at the fan inlet is the highest of the cycles observed, the thermal efficiency $\dot{Q}$ remains unaffected as there is no condensation effect of the water vapor in the air flowing through the air-water exchanger. Relatively smaller temperature differences, in particular T_out_a_—T_B_ and a reduction of the condensation effect, contribute to the fact that the highest observed COP_w_ does not exceed the value of 3.5 (Figure 25) and at lower solar radiation intensities they fall below the value of 1, which means that the heat storage process is completely impractical. 

The presented analysis shows that the work of the experimental greenhouse heat storage system is effective provided that a complex control system is used that could analyze not only the temperature difference, for example, T_in_a_—T_B_ but also the heat flux transferred to the accumulator. 

**Figure 26:**
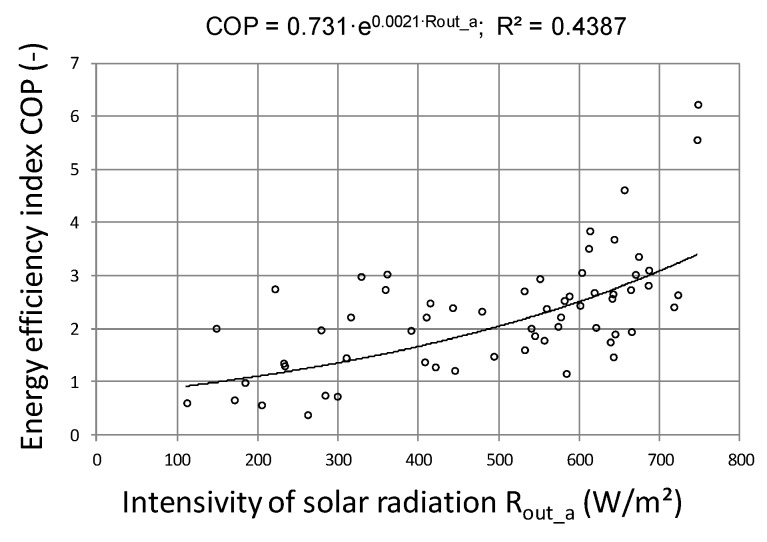
Energy efficiency of heat storage depending on the solar radiation flux.

**Figure 27:**
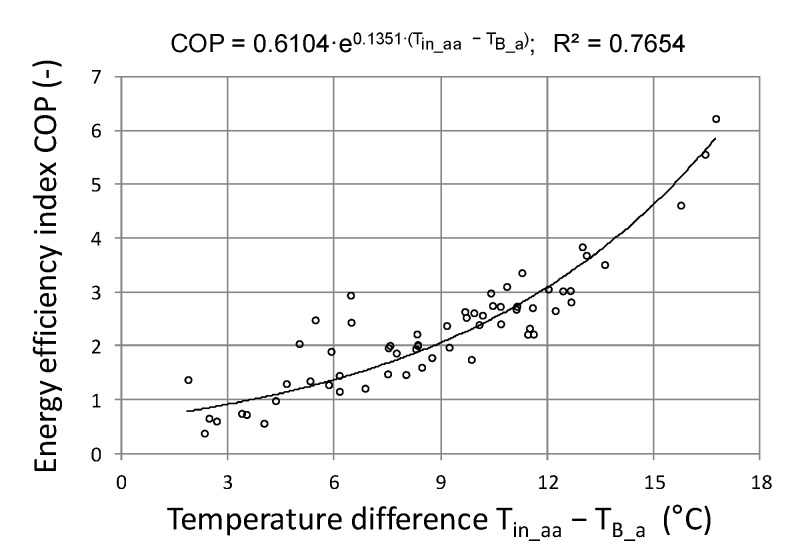
Energy efficiency of heat storage depending on the temperature difference.

Another aspect highlighted by the research was the calculation of the COP coefficient for the entire system work cycle, that is, from activation to shutdown. 62 work cycles were analyzed and were presented in energy efficiency charts depending on the solar energy R_out_a_ flux (Figure 26) and the temperature difference T_in_aa_−T_B_a_ (Figure 27). From the presented graphical relationship (Figure 26) one can notice a visible relationship between the COP coefficient and the solar radiation flux, however the developed model is poorly fitted. The impact of temperature difference T_in_aa_−T_B_a_ on the energy efficiency of the heat storage system is much stronger. The developed exponential model explains the phenomenon satisfactorily, which is confirmed by the R^2^ = 0.74. 

**Figure 28:**
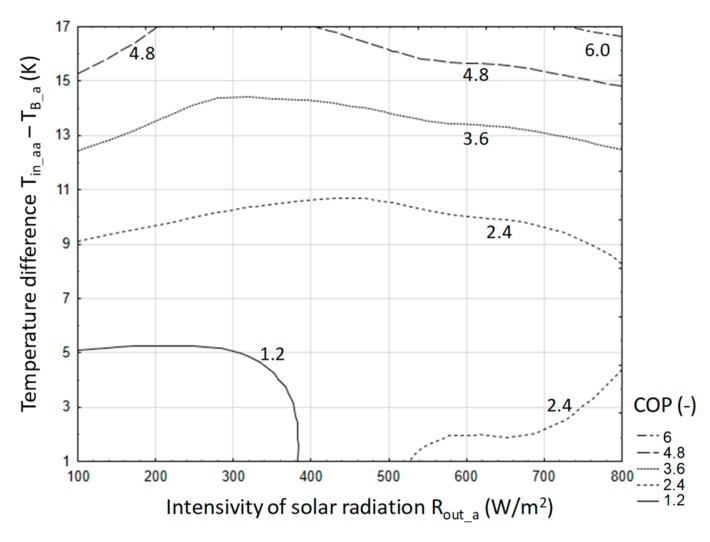
Energy efficiency of heat storage depending on temperature difference and solar radiation intensity.

In order to develop the relationship that best describes the phenomenon, estimation of multiple non-linear regression was performed. The developed model was presented an Equation (17) and its graphical interpretation in a contour plot (Figure 28).

(17) $$COP=1.38E^{-3}\cdot {\left(T_{in\_aa}-T_{B\_a}\right)}^{2.076}\cdot R_{out\_a}{}^{0.341}+1.004\;R^{2}=0.87,$$

where *T_in_aa_*—air temperature at the exchanger inlet (°C); *T_B_a_*—water accumulator temperature (°C); *R_out_a_*—intensity of solar radiation (W/m²).

For the developed model, the calculated error σ is at the level of 0.11.

Assuming that the COP of the storage process cannot be less than 1, then the relationship takes the form:

(18) $$1\leq 1.38\cdot 10^{-3}\cdot {\left(T_{in\_aa}-T_{B\_a}\right)}^{2.076}\cdot R_{out\_a}{}^{0.341}+1.004.$$

Inequality is always met if *T_in_aa_*>*T_B_a_*. However, assuming that the COP will be at a level greater than 3, which is dictated by the efficiency of the national power system, the relationship (18) can be reduced to the form (19):

(19) $$3\leq 1.38\cdot 10^{-3}\cdot {\left(T_{in\_aa}-T_{B\_a}\right)}^{2.076}\cdot R_{out\_a}{}^{0.341}+1.004\;.$$

If we use the approximation 0.341 ≅ 1/3 is used, then the relationship (19) can be reduced to the form (20):

(20) $$\;{\left(\frac{1446}{{\left(T_{in\_aa}-T_{B\_a}\right)}^{2.076}}\right)}^{3}\leq R_{out\_a}.$$

After parameterization of the relationship (19), it was established that the minimum difference *T_in_aa_* − *T_B_a_* should be greater than 11 °C, in order to achieve the assumed efficiency (COP = 3) with solar radiation 1000 W/m². It should be noted that the relationship (20) can be used to determine the boundary parameters of the heat accumulation system in a water accumulator for specific production and construction of a greenhouse or foil tunnel.

### 3.3. PCM Accumulator 

**Figure 29:**
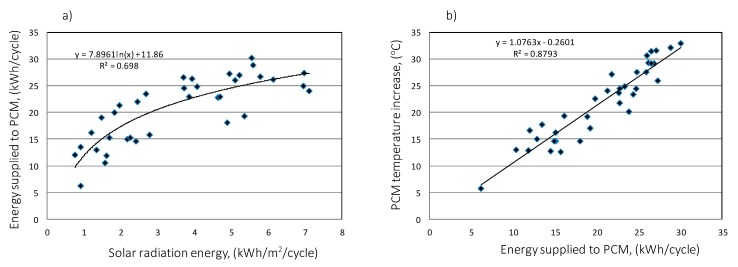
Energy stored in the accumulating bed depending on the duration of the charge cycle (**a**). Exhaust air enthalpy depending on the air enthalpy at the inlet of PCM accumulator (**b**).

The analysis shows a clear correlation between the charging time and the amount of heat stored in the accumulator bed (Figure 29a). However, by analyzing the individual accumulator charging cycles in detail, some of them indicate that the energy accumulated in paraffin can be twice as high despite the same cycle time, which is the result of different levels of solar radiation intensity and the ability to collect heat through the accumulator bed.

In the process of accumulator discharging, the amount of heat received depends on the difference in enthalpy of inlet and outgoing air from the accumulator (Figure 29b). There is a strong relationship between the energy input and output of the accumulator (correlation coefficient 0.93).

**Figure 30:**
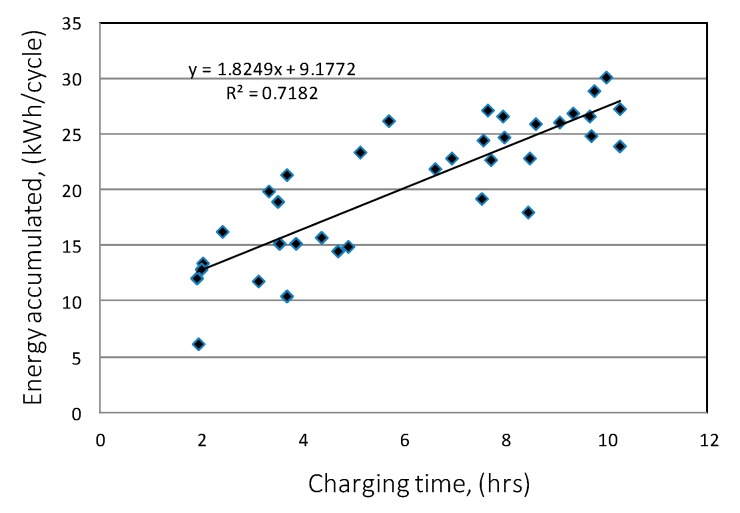
Useful energy as a function of discharge cycle time.

When analyzing the available amount of usable energy from the accumulator in its discharge cycle, it was found that the amount of this energy increases with the duration of the cycle. The graph in Figure 30 shows the relationship of these parameters. The correlation coefficient was 0.74.

**Figure 31:**
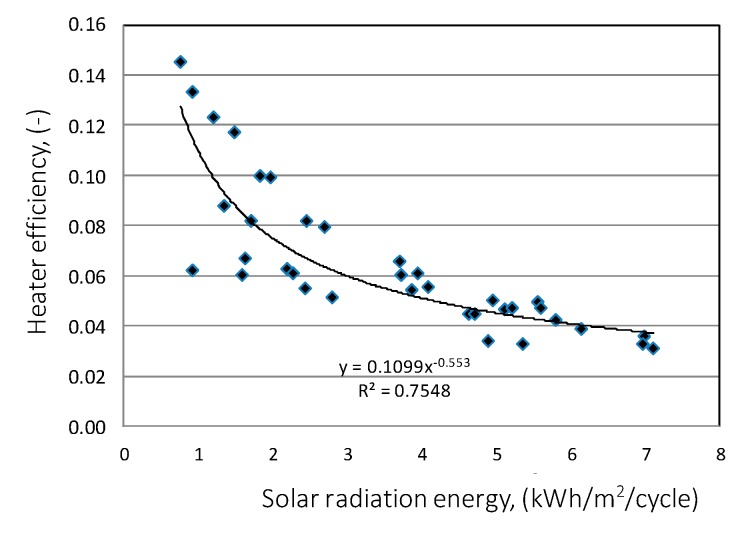
Efficiency of the PCM accumulator depending on the amount of usable energy.

Generally, a greater amount of usable energy available in a paraffin-filled accumulator occurs in the range of higher operating efficiency (Figure 31). However, there is no clear relationship between these parameters. This means that for a certain amount of available heat, the accumulator can work with varying degrees of efficiency. 

**Figure 32:**
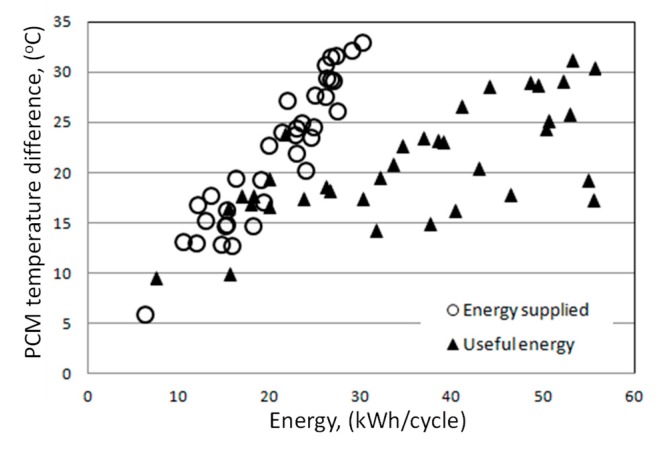
Difference in paraffin temperature as a function of supplied and usable energy of the PCM accumulator.

The analysis of the impact of supplied and usable energy on changes in the paraffin temperature in the accumulator bed shows that the energy supplied in the charging process gives a higher increase in bed temperature (Figure 32). This is the result of a larger temperature difference between warm air and paraffin. In addition, when using the difference in paraffin temperature, the range of temperature changes in the bed should be taken into account. This applies in particular to the use of such heat storage due to specific heat and/or specific heat and latent heat of the paraffin phase change.

This is clearly seen in the discharge process, where for a similar difference in paraffin temperature the useful energy can be up to three times higher.

**Figure 33:**
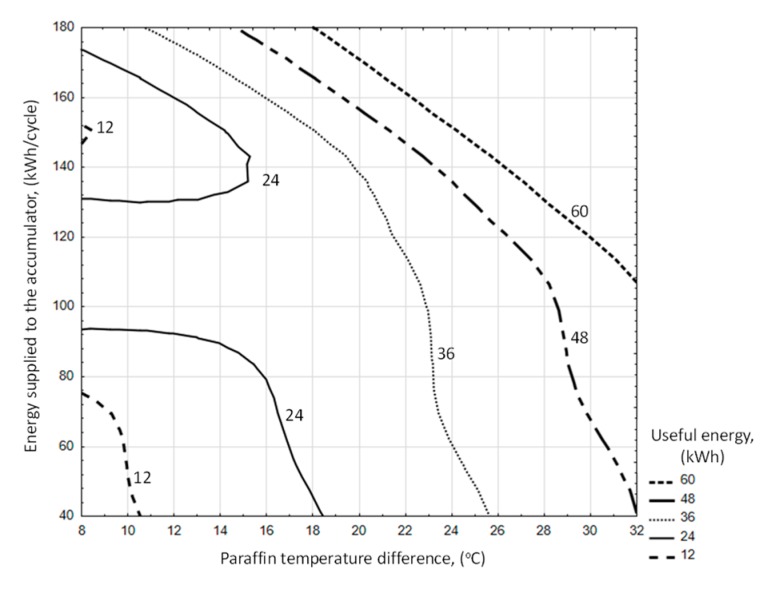
Useful energy in the heat storage process depending on the difference in paraffin temperature and energy supplied to the PCM accumulator.

The amount of usable energy available from an accumulator filled with paraffin (Figure 33) generally increases as a direct result of energy supplied for accumulation along with an increase in the temperature difference of the accumulator bed. Only in the range of low differences in paraffin temperature (from 8 to 16 °C) a different trend was observed. The equation determining the available useful energy (*Q_E_*) in the PCM accumulator cycle was determined as a function of the energy supplied to the accumulator and the increase in paraffin temperature, using a linear estimation with the quasi-Newton method. The significance of the parameters was set at a level less than 0.05.

Useful heat from the accumulator (kWh/cycle):

(21) $$Q_{E}=0.0493\cdot Q_{inlet}{}^{2}+0.00099\cdot ∆T_{paraffin}{}^{2}\;R^{2}=0.75$$

for the ranges of input variables:

$$43.99\leq Q_{inlet}\;\leq 178.3\;{}^{\circ}C\;9.5\leq ∆T_{paraffin}\;\leq 31.2\;{}^{\circ}C$$

**Figure 34:**
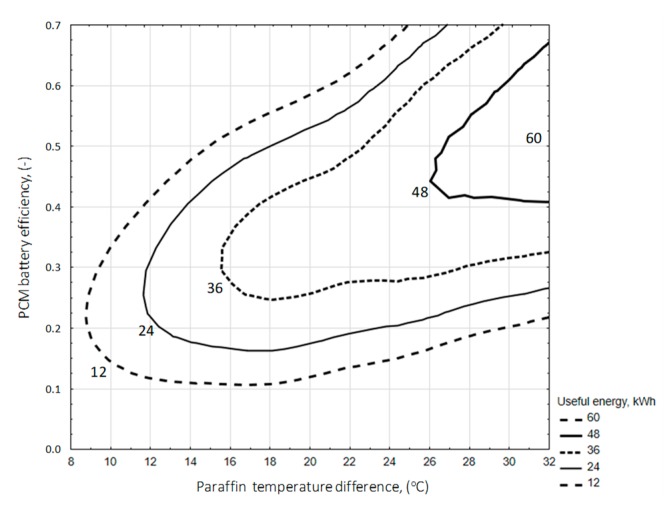
Useful energy in the heat storage process depending on the difference in paraffin temperature and heat storage efficiency.

The amount of usable energy available from a battery filled with paraffin (Figure 34) increases with increasing battery efficiency and with increasing temperature difference of the battery bed. The equation used to ascertain the available useful energy in the PCM accumulator cycle was determined as a function of accumulator efficiency (*E_ff_*) and paraffin temperature increase (Δ*t_paraffin_*), using a linear estimation with the quasi-Newton method. The significance of the parameters was set at a level less than 0.05.

Useful heat from the accumulator (kWh/cycle):

(22) $$Q_{E}=E_{ff}{}^{1.059}+28.996\cdot \;∆T_{paraffin}\;R^{2}=0.73$$

for the ranges of input variables:

$$11.8\leq E_{ff}\;\leq 60.5\;\%\;9.5\leq ∆T_{paraffin}\;\leq 31.2\;{}^{\circ}C.$$

**Figure 35:**
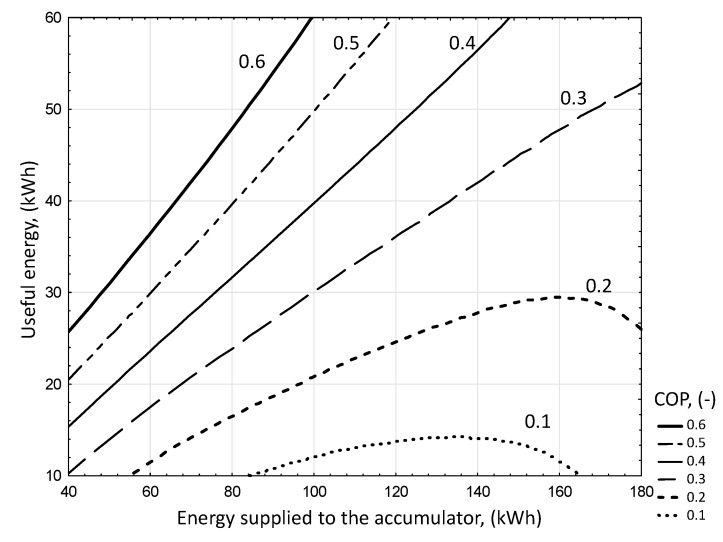
Changes in the COP coefficient taking into account energy supplied to the battery and useful energy.

Changes in the COP coefficient taking into account energy supplied to the battery and useful energy (Figure 35) increases with increasing battery efficiency and with increasing temperature difference of the battery bed. The equation for the efficiency of the PCM accumulator was determined in the function of energy supplied to the accumulator (*E_S_*) and usable energy (*Q_E_*), using a linear estimation with the quasi-Newton method. The significance of the parameters was set at a level less than 0.05.

Energetic efficiency index (COP) of the PCM accumulator (-):

(23) $$COP=-0.003\cdot E_{S}+0.009\cdot Q_{E}+0.381\;R^{2}=0.97$$

for the ranges of input variables:

$$44\leq E_{S}\;\leq 178.3\;kWh\;7.48\leq Q_{E}\;\leq 55.5\;kWh.$$

The efficiency of the PCM accumulator operation during the experiment was between 11.8 and 60.5%. Such a large variation resulted from the thermal range of the accumulator operation. The system achieved lower efficiency in the range of temperatures where only specific paraffin heat was used, whereas accumulator operation in the phase transition temperature range was characterized by higher efficiency.

### 3.4. Comparison of Analyzed Battery Types

The three types of heat accumulators investigated have been tested in low-temperature bed operation systems, that is, for a stone-bed accumulator 17.3 ÷ 37 °C, for a water accumulator 15 ÷ 37 °C and for a PCM 12.3 ÷ 55.2 °C. The given temperature range limits were extreme values that were reached during the experiment. As one can see, the temperature ranges of accumulator operation were similar, hence there were premises for comparative analysis, which resulted in the accumulator type being the preferred option in terms of heat storage. 

The energy efficiency coefficient of performance (COP) defined in the formulas (Equations (6), (11) and (15)) was used as the comparison criterion. This criterion takes into account the amount of heat accumulated in relation to the unit of electricity supplied to the system. With the efficiency of the electricity generation system, which in Poland is 35%, the desired energy effect should be achieved if the COP exceeds 3.0. The COP value results directly from the ratio of the primary fuel used to power plant turbines in relation to the direct use of this fuel for heating purposes. For example, if a power plant consumes 1 kWh of energy contained in the primary fuel within an hour and 0.35 kWh of energy is obtained when converting this energy into electricity, which means that that amount of electricity should contribute to the accumulation of 1kWh of primary energy. In the presented reasoning, the efficiency of the electricity transmission system and the primary fuel energy conversion system at the place of its use for the purposes of the heat accumulator was omitted. The above reasoning applies only to stone-bed and water accumulators, as in the case of the PCM battery, the power was realized by PV panels.

**Figure 36:**
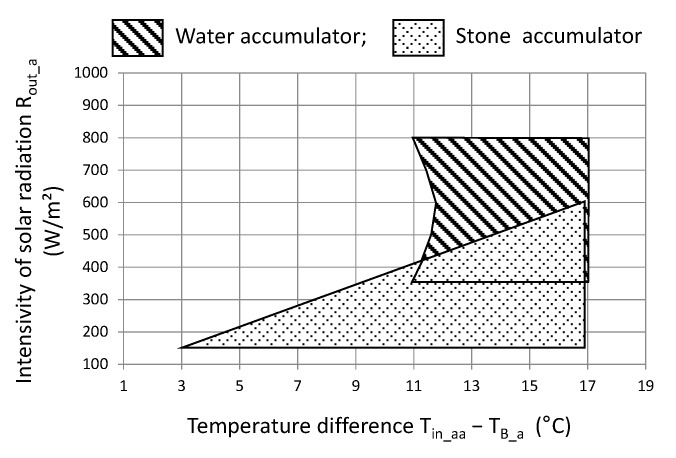
Recommended limits of work parameters of the analyzed accumulators for COP> 3.0.

With such a criterion, the work area can be determined in a rectangular coordinate system, that is, the difference between the inlet air temperature and the bed temperature and the intensity of solar radiation. The results are illustrated in Figure 36.

For a stone-bed accumulator, the desired energy effect can be obtained for a temperature difference (air inlet to the accumulator and bed temperature) in the range of 3 to 11 °C and average solar radiation intensity in the range from 150 to 600 W/m^2^. However, a water accumulator satisfies this condition if the average temperature difference is above 11 °C and the intensity of solar radiation is greater than 350 W/m². At the same time, the impact of solar radiation intensity is much smaller due to the thermal inertia of the entire accumulation system, especially during short periods of operation. However, the value of solar radiation intensity above 350 W/m² already indicates a significant share of direct radiation in the stream of solar energy reaching the tested object. Energy efficiency at COP = 3 is the lower boundary state required for the local energy system. For the purposes of a comparative assessment of the accumulators in question, the work area was also checked with an energy efficiency of not less than 5. In this respect, both stone-bed and water accumulators can be used. In the case of stone bed, such efficiency was obtained in several measurements, hence there are no grounds for safe inference for engineering practice. However, such a high efficiency can be achieved by a water accumulator, provided that the temperature difference is greater than 15 °C and the average intensity of solar radiation is above 450 W/m², which results in a full share of direct radiation.

The graphical analysis (Figure 36) also shows a practical premise that in the case of a stone-bed accumulator, this accumulator shows better efficiency at lower parameters, that is, temperature difference and solar radiation intensity. In turn, for a water accumulator, a higher temperature difference and a higher value of solar radiation intensity are recommended.

The PCM phase change accumulator has a much smaller COP and is not comparable with a stone-bed or water accumulator. The COP value for this type of accumulator does not exceed 0.6 and should not be used in thermal systems supporting the heating systems of horticultural facilities. Storage efficiency is influenced not only by the amount of energy acquired but also by the insulation of the tank walls [44]. The phase change accumulator works well for long-term heat storage. The tests [45] in which the PCM accumulator was used in the heating installation showed that it is possible not only to store excess heat but also to improve the efficiency of the entire system. In this application of the PCM accumulator, it is necessary to install heaters powered by PV panels. However, this configuration of the system solves the problem of coherence of the energy source, that is, the shift in energy supply and demand.

In both cases, adequate control is extremely important to obtain the maximum COP value. The efficiency of heat storage in a PCM accumulator can be increased by modernizing the material used for the construction of the accumulator and changing its geometry [46].

## 4. Conclusions

The research clearly shows that the accumulators used for heat storage (stone-bed and water), with rational control of their operation, can contribute to reducing the negative effects associated with heating facilities and furthermore, that the PCM accumulator, in its present format, should not be used for heat storage in the low temperature range. The most important conclusions resulting from the conducted analysis are—(1) the efficiency of operation of the stone-bed and the water accumulator is comparable in a wide range of parameters of the surrounding climate (thermal and solar); (2) the stone-bed accumulator has a profound energetic effect in the range of the average temperature difference (measured between the air inlet of the accumulator and the bed) from 3 to 11 °C and the average intensity of solar radiation in the range from 150 to 600 W/m^2^; (3) the water accumulator has a positive energetic effect in the range of higher operating parameters (temperature, insolation), that is, with a temperature difference above 11 °C and solar irradiance greater than 350 W/m^2^. 

Hence, the two analyzed types of accumulators can be recommended for horticultural practice, with the water accumulator preferred for short-term heat storage (e.g., when using it to heat water dedicated to plant irrigation) or to heat the greenhouse air at sunrise to prevent condensation of water vapor on slowly heated fruits or plants.
